# Design and Fabrication of Micro Saw Enabling Root-Side Cutting of Bone

**DOI:** 10.3390/mi14040856

**Published:** 2023-04-15

**Authors:** Pawan Pathak, Jack Fasano, Young-Cheon Kim, Sang-Eun Song, Hyoung Jin Cho

**Affiliations:** 1 Department of Mechanical and Aerospace Engineering, University of Central Florida, Orlando, FL 32816, USA; pawan.pathak@ucf.edu (P.P.);; 2 Research Center for Energy and Clean Technology, School of Materials Science and Engineering, Andong National University, Andong 36729, Republic of Korea

**Keywords:** micro saw, surgery, microfabrication, electroplating, osteoarthritis, osteochondral auto-graft transplantation (OAT)

## Abstract

A novel micro saw was fabricated using a combination of photolithography and electroplating techniques, resembling a miniature timing belt with sideways blades. The rotation or oscillation direction of the micro saw is designed to be perpendicular to the cutting direction so that transverse cutting of the bone is attainable to extract a preoperatively planned bone-cartilage donor for osteochondral auto-graft transplantation. The mechanical property of the fabricated micro saw obtained using the nanoindentation test shows that the mechanical properties of the micro saw are almost an order of magnitude higher than bone, which indicates its potential bone-cutting application. To demonstrate the cutting capability of the fabricated micro saw, an in vitro animal bone cutting was performed using a custom test rig consisting of a microcontroller, 3D printer, and other readily available parts.

## 1. Introduction

Osteoarthritis (OA) is one of the most common joint disorders with symptoms such as pain, inflammation, decrease in the range of motion, and reduction in activities of daily living [1]. Globally, OA affects nearly 528 million population, which is estimated to increase due to an increase in the aging population and obesity [2]. The study has shown that the causes of OA are being overweight, repetitive joint movements, aging, history of injury, low bone density, and muscle weakness, and it is a leading cause of disability in activities of daily living [3]. OA can occur in any joint in the body; it is most common in repetitive load-bearing joints because the flexible tissue (cartilage) at the end of the bone wears down, and the bone begins to change. Since the cartilage does not have blood vessels, nerves, or lymph, the healing of OA has been proven to be challenging [4]. Thus, treatment often requires surgical intervention. The typical treatment procedure involves removing the defect area and transferring the patient’s own bone and cartilage (osteochondral grafts) from the low load-bearing area, known as osteochondral auto-graft transplantation (OAT) [5]. Since this procedure uses the patient’s own tissue, it eliminates the risk of infectious disease transmission and helps treat smaller osteochondritis dissecans lesions [6]. In addition, the defective bone can be filled with mature hyaline cartilage; thus, the cartilage and bone can be treated simultaneously [7].

**Figure 1:**
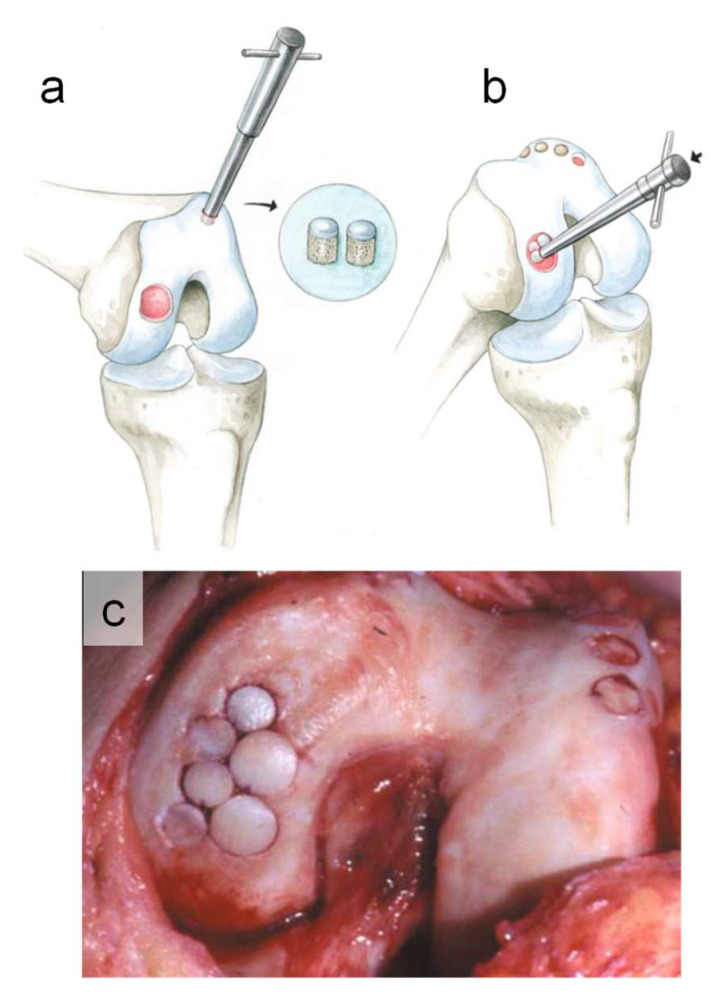
Illustration of OAT surgery technique showing (**a**) defect debridement and donor graft harvesting, (**b**) implantation of the donor grafts into the defect, and (**c**) picture of the bone showing donor grafts implantation into the defect. The figure is reproduced from ref. [16].

Mosaicplasty is a well-known technique since small cylindrical autografts can easily be extracted and then can fill any defect shape. Multiple grafts are able to resurface 80–100% of the damaged area [8], but studies have shown that having a gap between cartilages can result in poor histologic properties of the cartilage [9]. The current tool for removing the donor graft in OAT surgery is a manually inserted hollow cylindrical rod that is used to break the base of the donor graft [10,11], as shown in Figure 1. After removing the donor graft, its base is cut to implant into the defect site [12]. The technological limitation of this procedure is precisely filling the prepared defect area with multiple healthy osteochondral donor grafts. To overcome this limitation, a bone-cutting mechanism that can harvest a predetermined shape and length of the donor graft is required. The working mechanism of the proposed OAT surgery technique is described in detail in the previous work [13,14]. K-wires or cylindrical hollow drill bits can be used to make the cylindrical cut of the donor graft [15]. For the transverse cutting of the donor graft, a unique micro saw is required, which is not currently available, and this work is focused on fabricating a micro saw for this application. The minimum requirements of the micro saw for this application are the width of the micro saw to be less than 1 mm and the micro saw capable of in situ transverse cutting of donor graft. We hypothesize that such a micro saw can be fabricated using micro electroplating and photolithography, and the fabricated micro saw will be strong enough to cut the bone. The advantages of the proposed technique are a decrease in bone stress (donor site), precise cut, and optimal use of the donor graft.

Advancements in engineering have made available numerous micromachining technologies that have been utilized in fabricating medical devices [17,18,19]. Electroplating can be employed as one of the micromachining technologies when combined with photolithography to make miniaturized devices with various types of metals such as nickel, gold, silver, copper, and their alloys [20,21]. Electrical, mechanical, and magnetic characteristics, as well as chemical homogeneity of the metal layer, are the main parameters to control for a high-performance component [22,23]. Therefore, it is critical to control, optimize, and standardize the fabrication parameters. Electroplating with the modulated DC (direct current) at a specified duty cycle, known as pulse electroplating, is popular for micro electroplating [24]. In pulse electroplating, elimination of the hydrogen embrittlement due to the depletion of cation species in the pulsating diffusion layer improves homogeneity, decreases porosity, and enhances adhesive strength [25,26]. The enhancement of the mechanical characteristics of nickel was also observed when the size of the electroplated nickel structure was reduced [27]. This favorable scaling effect, biocompatible nature, and its native mechanical properties make the electroplated nickel a superb candidate for the micro saw blade.

This research aims to develop a micro saw using a biocompatible material that is capable of precisely removing the defected area and root-side cutting of the donor graft. In order to achieve these goals, the nickel micro saw with transversal blades was designed and fabricated, resembling a miniature timing belt with sideways blades using an electroplating process combined with two-step photolithography. The micro saw was designed with a simple structure without a mechanical connector to maximize its lifetime. The mechanical, morphological, and chemical properties of the fabricated micro saw were studied in detail.

## 2. Materials and Methods

### 2.1. Chemicals

Acetone, isopropanol, nickel chloride, and methanol were obtained from Sigma-Aldrich (St. Louis, MO, USA). AZ 40XT photoresist and MIF 300 developer were obtained from Microchemicals GmbH (Nicolaus-Otto-Straße, Ulm, Germany). Nickel sulfamate electroplating bath solution was purchased from Technic Inc., Cranston, RI, USA. All these chemicals were used without additional purification.

### 2.2. Design of Micro Saw

**Figure 2:**
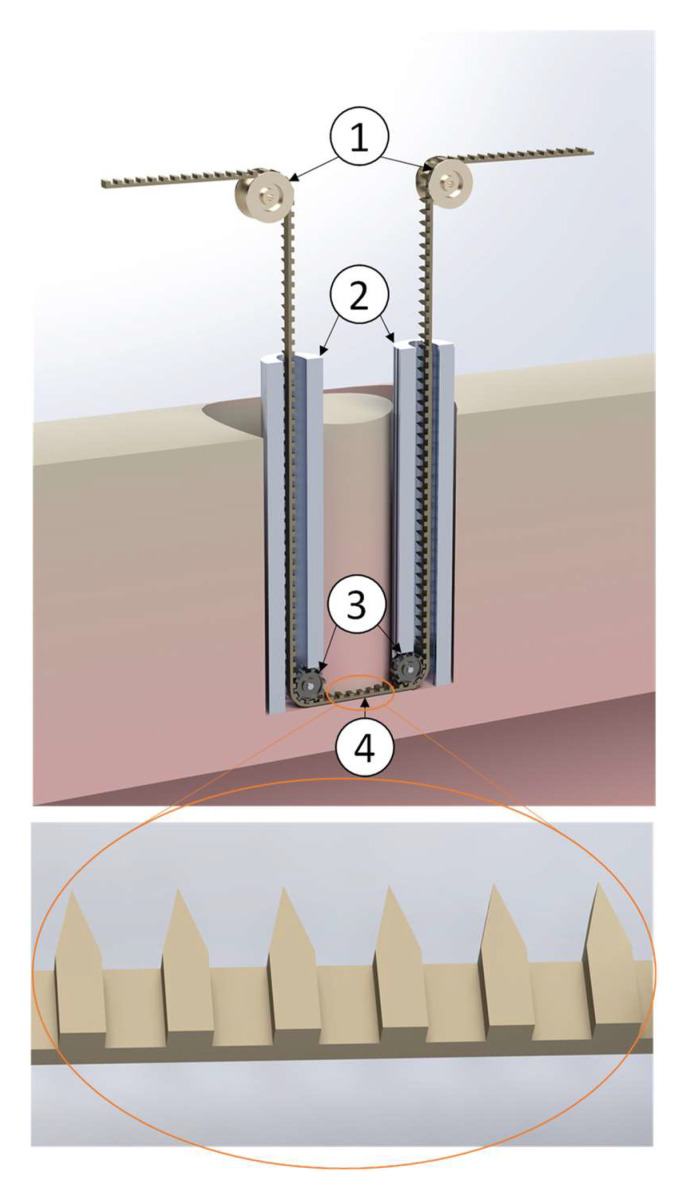
CAD model of donor root-side cutting mechanism showing (1) micro chainsaw guides inserted in donor profile (drilled), (2) pulley, (3) saw driver, and (4) micro saw (bottom) zoomed view of micro saw in action.

The schematic illustration of the design of the micro saw and its working mechanism is presented in Figure 2. In order for precise custom defect removal and autograft donor harvesting, a novel micro saw design is required, which is currently unavailable. Figure 2 illustrates the micro saw operation mechanism in which the saw operates through guide tubes. The micro saw guide will ensure that the micro saw will not damage the bone wall during its operation. The two main requirements for such an operation are: (a) the micro saw is small enough to travel inside the saw guide at ~1 mm diameter; and (b) the micro saw blades are perpendicular to the travel loop. This work mainly deals with designing and fabricating the prototype micro saw that meets the above requirements. The micro saw was designed in such a way that it has blades perpendicular to the direction of the motion, which has the advantage of root-side cutting of the bone. The micro saw will resemble the micro timing belt, with the blade perpendicular to the gear teeth. It was also designed to be single-use to prevent the risk of cross-contamination and infection. The fabricated prototype includes the micro saw, and the micro saw driver.

### 2.3. Fabrication of Micro Saw

**Figure 3:**
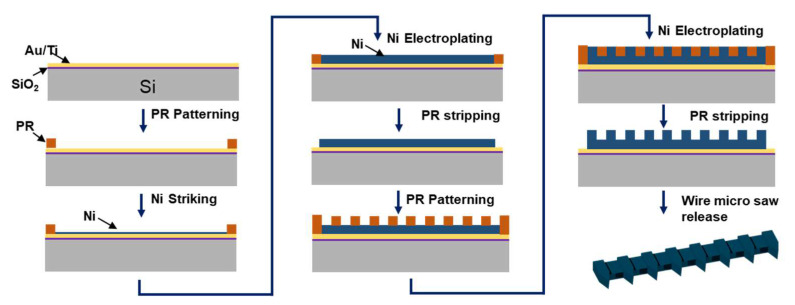
Schematic illustration of micro saw fabrication process.

The micro saw with transversal blades was fabricated by photolithography in combination with electroplating. Multiple micro saws were fabricated using a 3-inch oxidized silicon wafer. Figure 3 shows the schematic illustration of the fabrication process. First, 10 nm of Ti and 100 nm of Au thin film were deposited using an e-beam deposition. Then, the AZ 40XT photoresist was spin-coated at 800 rpm for 30 s, followed by baking at different temperatures (75 °C for 120 s, 105 °C for 120 s, and 125 °C for 180 s). Later, the substrate was exposed to UV using a mask aligner (EVG 620 mask aligner, St. Florian am Inn, Austria) and developed using AZ300MIF to create the patterns. The photoresist pattern opening is used as the mold to electroplate nickel. The photoresist pattern and the electroplating conditions were optimized based on uniformity and morphology. The electroplating parameters such as current, temperature, and electrolyte composition are crucial in controlling the grain size, uniformity, composition, and mechanical properties. The nickel seed layer was deposited by connecting a patterned wafer as a cathode and a nickel plate as an anode. The 10 mA/cm^2^ current density was applied for 5 min using nickel chloride as the electrolyte. Nickel striking was used to clean the base material and deposit a thin nickel layer for good adhesion. After depositing the nickel seed layer, the nickel was electroplated using the commercial nickel sulfamate bath solution at 55 °C. The electroplating was performed using the pulse current density of 10 mA/cm^2^ with a duty cycle of 50% (10 ms off, 10 ms on) for 7 h. Nickel is electroplated slowly in the photoresist mold until the desired thickness is achieved. The photoresist layer was then stripped using acetone. After fabricating the bottom layer, micro saw teeth (top layer) were microfabricated using a similar technique as the one used to fabricate the base layer. The AZ 40XT photoresist was spin-coated on the surface, and nickel was baked at the temperature described above. The photomask of the top layer was manually aligned with the base layer and exposed to UV using EVG aligner. The pattern was developed in the developing solution and electroplated to create the top layer of the micro saw. The micro saws were released from the silicon substrates, etching the underneath gold layer. The potassium iodide solution was used as a gold etchant.

### 2.4. Materials Characterization

The surface morphologies of the samples were probed using a Zeiss ultra 55 scanning electron microscope (SEM) system (Carl Zeiss SMT GmbH, Oberkochen, Germany) operated at 5 keV. Samples were positioned at a 5 mm working distance to take SEM images. EDX (energy dispersive X-ray) microanalysis of the samples was performed at an acceleration voltage of 15 keV and at a working distance of 15 mm. The fabricated micro saw, and the micro saw driver were profiled using 3D optical microscope images (Keyence VHX-2000, KEYENCE Corp., Osaka, Japan). The mechanical properties of the samples were evaluated using a KLA iMicro nanoindenter system equipped with a three-sided Berkovich indenter. The tests were conducted on the upper side of the samples, at least 20 µm away from the edges, with a maximum indentation depth of 1 µm, which is less than one-tenth of the sample thickness. A total of 25 nanoindentation data points were obtained at each location of the sample, with a strain rate of 0.05 s^−1^, and thermal drift lying within 0.05 nm/s.

## 3. Results and Discussion

### 3.1. Morphology and Chemical Analysis

**Figure 4:**
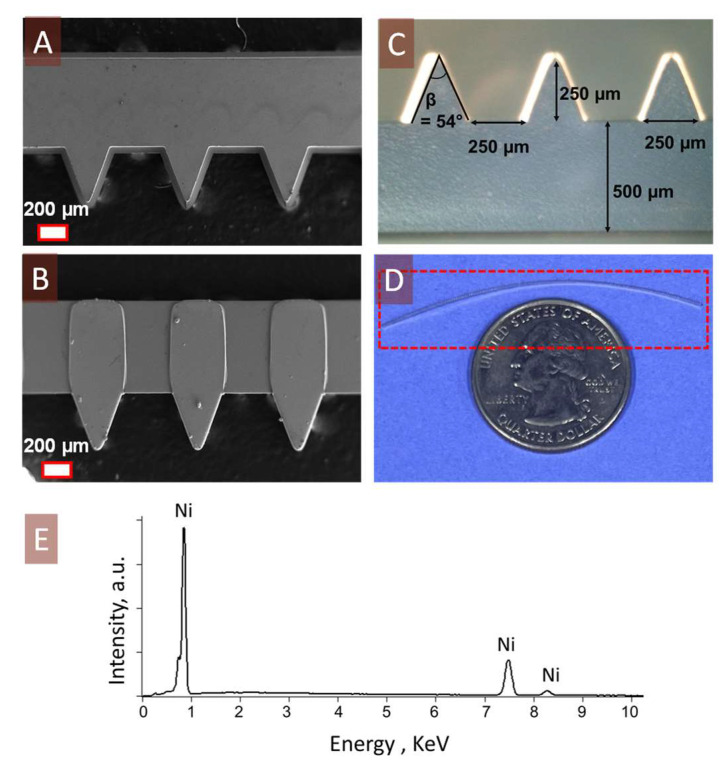
(**A**,**B**) SEM images of the fabricated micro saw bottom, and top view, (**C**) 3D optical microscope image showing micro saw tooth anatomy, β = wedge angle, (**D**) photograph of the fabricated micro saw size comparison with the US quarter coin, and (**E**) EDX analysis of the device.

To analyze the morphology of the fabricated micro saw, SEM and 3D optical microscopy were performed. Figure 4A,B show SEM images of a top and bottom view of the micro saw. It is clear from the SEM images that well-defined high-resolution micro saws were fabricated. The SEM images (Figure 4A,B) show that the micro saw’s cutting direction is perpendicular to its travel direction. The unique design of the fabricated micro saw will enable root-side cutting of the bone. The thickness of the base layer and micro saw tooth thickness measured using a 3D microscope were 85 ± 4 µm and 63 ± 3 µm, respectively (Figure S1). Figure 4C shows a 3D optical microscope image showing micro saw tooth anatomy. The saw has a tooth height of 250 microns, a tooth wedge angle of 54 degrees, a space of 250 microns between teeth, and a saw width of 500 microns. The pitch of the micro saw is 4 teeth per mm with an average thickness of 85 ± 4 µm. Micro saw anatomy was designed based on the requirement for symmetry, which is needed for optimal oscillating cuts [28]. Figure 4D shows the photograph of the fabricated micro saw, and the dimensions were compared with the US quarter coin. The dimension of the micro saw was 5 cm × 750 µm × 148 µm. EDX (energy dispersive X-ray) microanalysis of the micro saw presented in Figure 4E showed distinct peaks at 0.85 KeV and 7.47 KeV, which correspond to the L_α_ and K_α_ lines of nickel. The result confirms that high-purity nickel is electroplated on the Au/Ti deposited silicon wafer.

### 3.2. Mechanical Analysis

**Figure 5:**
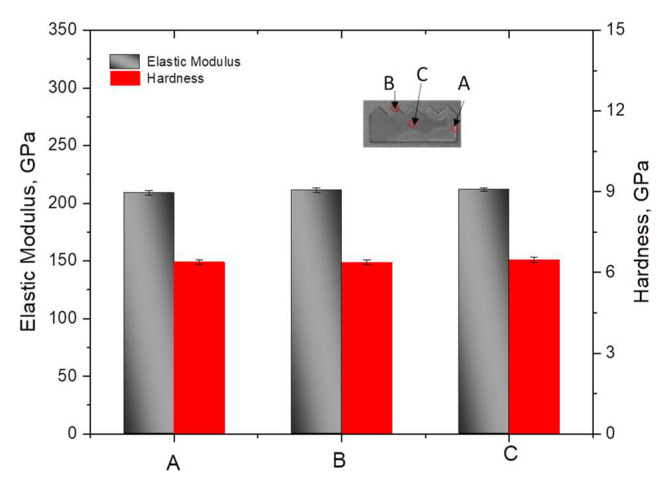
Elastic modulus and hardness of micro saw at areas A, B, and C, obtained using nanoindentation.

Figure 5 presents the elastic modulus and hardness values obtained from nanoindentation force-depth curves using the Oliver–Pharr method [29] at three different areas (A, B, C) on the surface of the micro saw. The testing areas were selected as representative spots on the sample, with A located near the left or right side, B near the top or blade of the sample, and C in the middle. The elastic modulus values of the three spots were 211 ± 2.31 GPa, 210 ± 1.79 GPa, and 212 ± 1.51 GPa, respectively. The corresponding hardness values were 6.76 ± 0.09 GPa, 6.87 ± 0.10 GPa, and 6.63 ± 0.07 GPa, respectively. These results show less than 1% deviation in the mechanical properties, indicating that homogeneous electroplating was conducted on the overall specimen. The absolute values of elastic modulus and hardness obtained in this study are consistent with previous researchers [30,31], indicating that the mechanical properties of the micro saw are within the expected range for the material. The average elastic modulus and hardness of the human bone are in the range of 11 to 22 GPa and 0.2 to 0.7 GPa, respectively [32,33,34]. The mechanical properties of the fabricated micro saw are almost an order of magnitude higher than that of human bone. In addition, high hardness value of nickel provides good wear resistance for sliding contacts, indicating the micro saw’s bone cutting capability [35].

### 3.3. Bone Cutting Analysis

**Figure 6:**
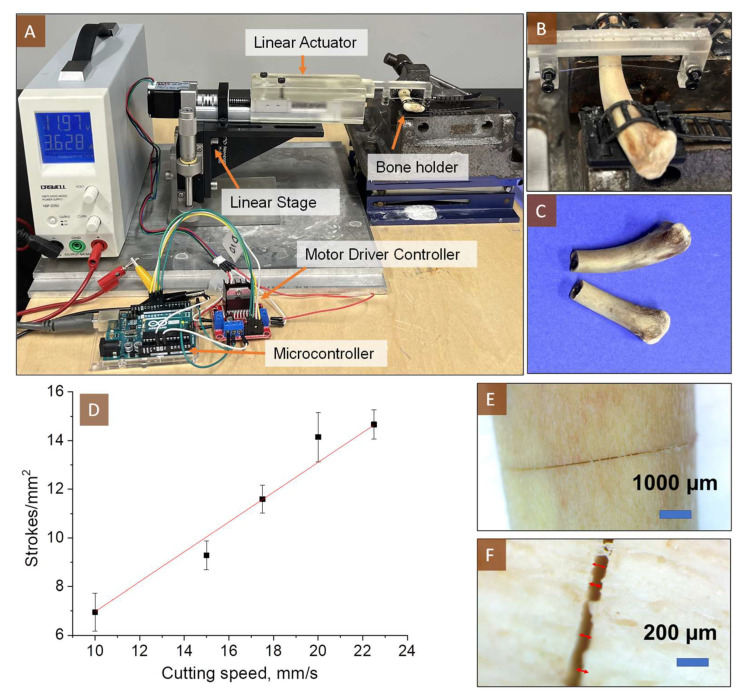
(**A**) Bone cutting setup, (**B**) a zoomed view of micro saw in action, (**C**) a picture of the bone showing uniform cutting, (**D**) number of strokes per unit area vs. cutting speed, and (**E**,**F**) optical microscope images of a bone cut at different magnification showing the uniform sub-100-micron wide cut.

The CAD file of the micro saw blade holder and the setup were designed using solid works (Figure S2). The designed CAD models were printed using a 3D printer (Formlab 3B+). The home-built cutting setup was made using FUYU FSK30J Linear Actuator, Arduino Uno microcontroller, Newport linear stage, and L298N Motor Drive Controller Board. All these parts were assembled using 3D-printed structures. Later, the required electrical connection was made to power and control the step motor of the linear actuator. After building the setup, the Arduino microcontroller was programmed (Code S1) to move the linear actuator in a cutting motion at the desired degree of cutting speed. It is important to examine the performance of the fabricated saw for cutting the bone. To demonstrate the cutting capability of the fabricated micro saw, chicken bone was clamped and cut using the micro saw. The bone cutting setup and fully cut bone are shown in Figure 6A–C. The feed rate was manually controlled using a micrometer of the linear translation stage (Newport 433 Precision Linear Translation Stage). The maximum feed rate for the fabricated micro saw was 750 µm/min at the speed of 10 mm/s. If the feed rate exceeds 750 µm/min, the blade edge deformation and twisting of the saw were observed. The effect of the saw speed on strokes per mm^2^ was evaluated at a constant feed ratio of 750 µm/min. A linear correlation was observed between the strokes/mm^2^ and the saw cutting speed, as shown in Figure 6D. After analyzing the cutting performance, micro saw wear was morphologically examined using a microscopic technique. Since the micro saw was designed to be single-use, wear characteristics were examined before and after its single-use. Although bone debris (Figure S3) was found on the micro saw, no significant mechanical damage was observed, as predicted by the hardness testing results. In addition, to study the width and uniformity of the micro saw cut, an optical microscope image of the halfway-cut bone (Figure 6E,F) was analyzed. The width of the micro saw cut was 88 ± 3 µm with a smooth bone cross-section. These testing results suggest that the fabricated micro saw can be potentially used for root-side bone cutting.

## 4. Conclusions

The novel micro saw with integrated micro saw driver teeth was successfully fabricated using photolithography and electroplating. One of the critical parameters is the mechanical strength of the fabricated micro saw; the indentation test of the fabricated micro saw shows the hardness and the elastic modulus of 211 GPa and 6.75 GPa, respectively; these values are an order of magnitude higher than that exhibited by the human bone. For the root-side cutting of the donor without damaging the adjacent bone or cartilage, the micro saw needs to travel through a pair of guide tubes along the donor profile cut (1 mm or less). The fabricated micro saw with the integrated micro saw driver teeth is 750 µm in width with 148 µm thickness which is small enough to meet such a requirement. The width of the straight cut on an animal bone as small as 88 µm was demonstrated. The rotation or oscillation direction of the micro saw is designed to be perpendicular to the cutting direction; the fabricated micro saw can provide transverse cutting of the bone to extract a preoperatively planned bone-cartilage donor. This paper investigates the micro saw design and fabrication, providing future research directions for a robotic automated osteochondral tissue harvesting method. The proposed robotic tissue harvesting method can remove virtually any shape and size of complex donor grafts for autograft transplantation to treat severe osteoarthritis.

## Data Availability

The data that support the findings of this study are available from the corresponding authors on reasonable request.

## Supplementary Materials

The following supporting information can be downloaded at: https://www.mdpi.com/article/10.3390/mi14040856/s1, Figure S1: 3D optical microscope images of saw and saw driver; Figure S2: CAD model of 3D printed part used to build test setup; Code S1: Arduino code used for controlling the linear actuator; and Figure S3: Optical microscope images of saw blade tooth surface before and after cutting bone.
