# Supplementary material for: Design and Fabrication of Micro Saw Enabling Root-Side Cutting of Bone
Source: Micromachines (Basel). 2023 Apr 15;14(4):856. doi: 10.3390/mi14040856 (PMC10145077; doi:10.3390/mi14040856)
Supplement: Supplementary file 1 [file micromachines-14-00856-s001.zip › micromachines-2311136-supplementary.pdf]

Supplementary Material

Design and Fabrication of Micro Saw Enabling Root-side Cutting of Bone

Pawan Pathak[1], Jack Fasano[1], Young-Cheon Kim[2], Sang-Eun Song[1,*], Hyoung Jin Cho[1,*]

[1] Department of Mechanical and Aerospace Engineering, University of Central Florida, Orlando, FL 32816, USA.

[2] Research Center for Energy and Clean Technology, School of Materials Science and Engineering, Andong National University, Andong 36729, Republic of Korea

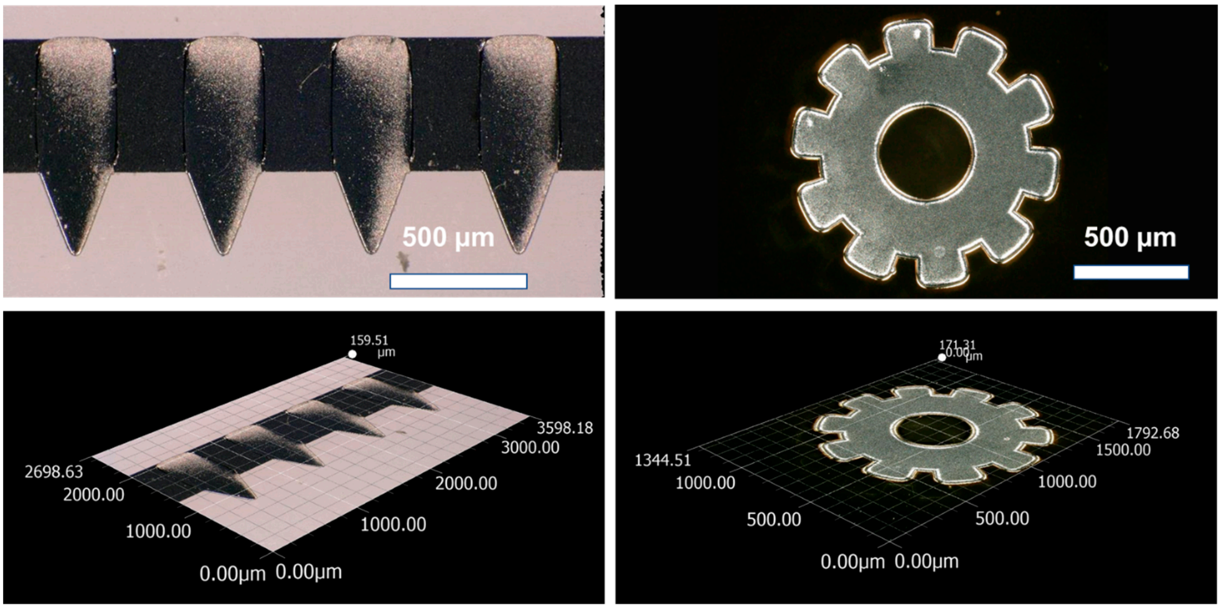

Figure S1. 3D optical microscope images of micro saw and micro saw driver.

.

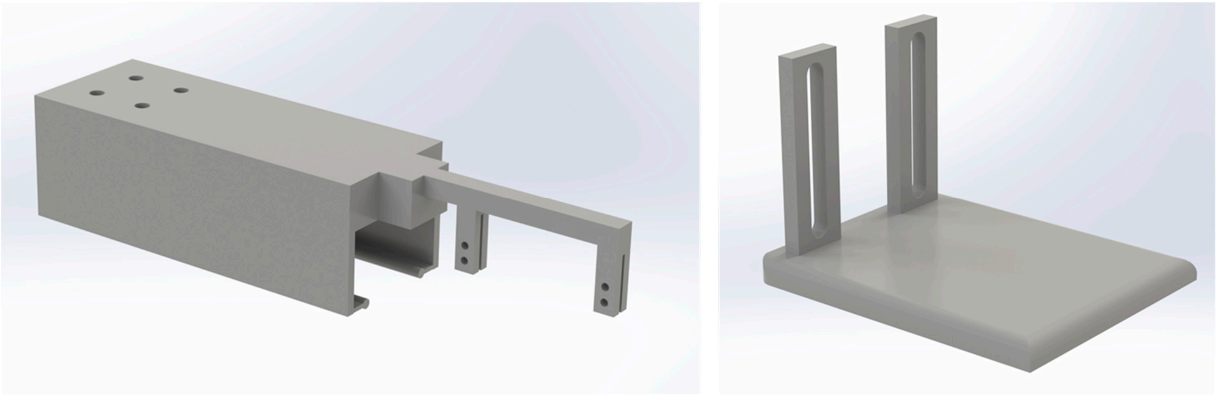

Figure S2. CAD model of 3D printed part used to build test setup.

Code S1: Arduino code used for controlling the linear actuator.

```
// define pins for stepper motor
const int coil1 = 8;
const int coil2 = 9;
const int coil3 = 10;
const int coil4 = 11;

// define sequence of steps for clockwise rotation
const int stepsCW[4][4] = {
  {HIGH, LOW, LOW, HIGH},
  {HIGH, LOW, HIGH, LOW},
  {LOW, HIGH, HIGH, LOW},
  {LOW, HIGH, LOW, HIGH}
};
// define sequence of steps for counter-clockwise rotation
const int stepsCCW[4][4] = {
  {LOW, HIGH, LOW, HIGH},
  {LOW, HIGH, HIGH, LOW},
  {HIGH, LOW, HIGH, LOW},
  {HIGH, LOW, LOW, HIGH}
};
int x=0;
// define number of steps per revolution
const int stepsPerRev = 2000;

void setup() {
  // set the coil pins as outputs
  pinMode(coil1, OUTPUT);
  pinMode(coil2, OUTPUT);
```

```
  pinMode(coil3, OUTPUT);
  pinMode(coil4, OUTPUT);
  Serial.begin(9600);
}
void loop() {
if (x==0){
delay(1000);
}
  // rotate the motor one revolution  clockwise
  for (int i = 0; i < stepsPerRev; i++) {
    int step = i % 4;
    digitalWrite(coil1, stepsCW[step][0]);
    digitalWrite(coil2, stepsCW[step][1]);
    digitalWrite(coil3, stepsCW[step][2]);
    digitalWrite(coil4, stepsCW[step][3]);
    delayMicroseconds(1000); // adjust speed of rotation by changing delay time
  }
  x++;
  Serial.print("Strokes");
  Serial.println(x);
  delay(1000);
  // rotate the motor one revolution  counter-clockwise
  for (int i = 0; i < stepsPerRev; i++) {
    int step = i % 4;
    digitalWrite(coil1, stepsCCW[step][0]);
    digitalWrite(coil2, stepsCCW[step][1]);
    digitalWrite(coil3, stepsCCW[step][2]);
    digitalWrite(coil4, stepsCCW[step][3]);
    delayMicroseconds(1000); // adjust speed of rotation by changing delay time
  }
  x++;
  Serial.print("Strokes");
  Serial.println(x);
  delay(100);

}
```

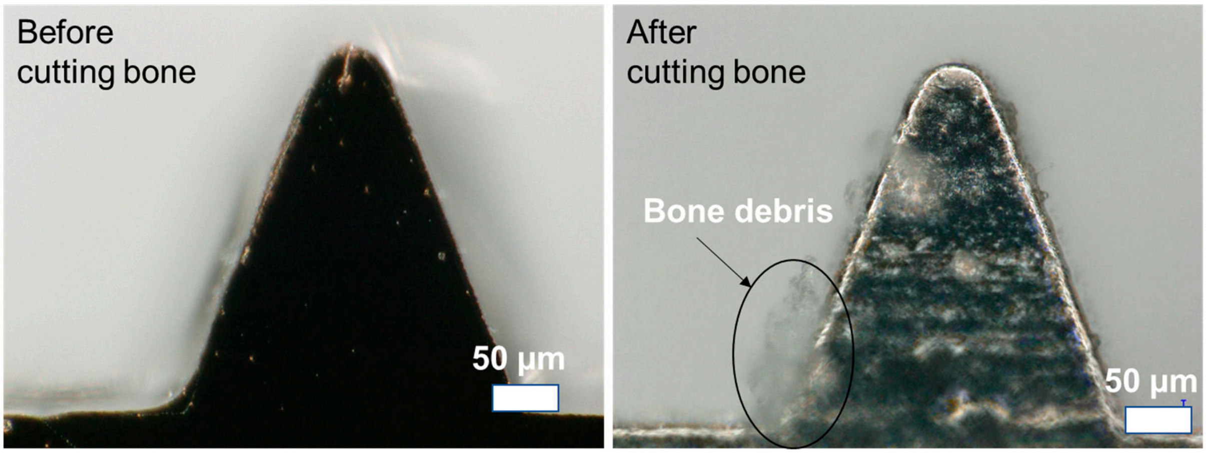

Figure S3: Optical microscope images of saw tooth blade surface before and after cutting bone.
